# Lord Kelvin at St. George's Hospital

**Published:** 1904-11-12

**Authors:** 


### LORD KELVIN AT ST. GEORGE'S HOSPITAL.

Last week Lord Kelvin presided at the annual distribution of prizes to the students of St. George's Hospital Medical School. A large number of people, including members of the medical and surgical staff, students, and others assembled in the Board Room of the hospital where the distribution took place.

Lord Kelvin, after presenting the prizes, congratulated the unsuccessful as well as the successful students. Those who were not prizemen, he said, had, nevertheless, received the benefit of the valuable teaching afforded by the School. The education they were all having, or had already had, was fitting them for earning an honourable livelihood in a splendid profession. That was more valuable than prizes could signify. Modern medical men must be scientific men, and, more, they must be philosophers. They were all being made philosophers. They were taking clinical duty, and that would teach them, as beginners at all events, to become philosophers. Their long and difficult course of study embraced dynamics and mechanics, physics, medicine and surgery, bacteriology, chemistry, physiology, anatomy, and pathology. But there was an additional subject which was not enumerated amongst those already mentioned, and that was human nature. For this study no prizes were given to the students. But, throughout their course, they were all students of human nature, and that went far beyond dead matter. The hocus-pocus of electricity, or viscous fluids, could not make a living cell. Huxley, Hooker, and others had thoroughly investigated the subject, but no artificial process could make living matter out of dead matter. The phenomena of life and the phenomena of death were subjects which medical men would meet with every day, often too sadly, in their practice, whether in hospital or in the general world. And every practitioner had to administer spiritual consolation to his patients. Let it, he said, not be thought that he meant to encroach on theological questions, but if practitioners would let their natural feelings prompt them in dealing with patients, they could not go wrong, and thus they would be spiritual aids. Cheerfulness and kind words were invaluable. A sick man lying with a broken leg will anxiously wait all day for an expected kind look or word from a doctor or nurse.

Mr. Timothy Holmes, one of the consulting surgeons of the hospital, and an old pupil of Lord Kelvin's, proposed a vote of thanks to the chairman. Dr. Ewart, senior physician, seconded the motion, and the proceedings then terminated with hearty thanks to Lord Kelvin.

The list of prizemen, each of whom was greeted with much applause as he received his award, is as follows:—William Byam, the Brackenbury Prize in Medicine, the Brackenbury Prize in Surgery, and the Thompson Medal; James Sholto Cameron Douglas, the Webb Prize in Bacteriology; Frank Lidbroke Cope, the Treasurer's Prize, and a Certificate of Proxime Accessit for the Brodie Prize; James Aubrey Torrens, Sir Benjamin Brodie's Clinical Prize in Surgery; Harry Gordon Webb, the Henry Charles Johnson Prize in Anatomy, the Pollock Prize in Physiology, and Prize for Proficiency in Anatomy, Physiology, and Physiological Chemistry; Alfred William Moore, Sir Charles Clarke's Prize; Valentine Henry Blake, Prize for Proficiency in Medicine, Surgery, Obstetric Medicine, and Pathology; Evelyn Henry Bardens Oram, a Certificate of Proficiency in Medicine, Surgery, Obstetric Medicine, and Pathology; Claude Howard Stanley Frankau, Prize for Proficiency in Organic Chemistry, Anatomy, and Physiology, and Physiological Chemistry, and a Certificate of Honour in Anatomy; Jose Leighton Houlton, Prize for Proficiency in Chemistry, Biology, and Anatomy; Paul Walde, an Entrance Scholarship in Arts of the value of £100; Eliezer Coplans, an Entrance Scholarship in Arts of the value of £50.

